# Removal of a Neuromatous Tumor

**Published:** 1868-03

**Authors:** George D. Slocum

**Affiliations:** Wyoming, N. Y.


### Correspondence.

### Removal of a Neuromatous Tumor.

BY GEORGE D. SLOCUM, M. D., WYOMING, N. Y.

Dr. Miner:—In the December issue of your Journal I noticed the remarks concerning your extirpating two tumors of nerve, and the perfect success of the treatment in relieving pain, and I therefore send you the following account of a case which recently came under my observation:

The patient, Mr. Edwin Stanley, aged 70, consulted me over a year since, in regard to a tumor situated on the inner side of the leg, just above the knee-joint. I advised removal, to which operation he consented on the 15th of January last. The tumor had existed for over eight years, gradually increasing in size, and measured two and one-half inches in length and one and one-half in breadth, ovoidal in shape, firm, and inelastic, and exquisitely tender on pressure. He could assign no cause for its occurrence, having never received any external injury—as wound, blow, or bruise. He experienced much inconvenience in walking, though no decided pain in the tumor itself, except from pressure, but down the inner side of the leg and foot he suffered exceedingly severe, sharp, and stinging neuralgic pain, felt most at night, and in proportion to the amount of exercise during the day. An incision three inches in length was made over the tumor. The tissues separated, when the filaments of the internal saphenous nerve were found separated, about two-thirds spread out upon the posterior surface. These immediately uniting in a common trunk at the lower extremity of the tumor. The tumor was easily removed after dividing the nerve above and below. The internal mass was quite dense, nearly white, and homogeneous. The wound was closed by sutures, and a firm compress applied beneath the part, and treated in the ordinary manner. It healed nicely, and the sutures were removed on the sixth day. The relief from pain was complete, he resting better at night than he had for years before. The third day he complained of the neuralgic pain returning, and he suffered severely therefrom, but that gradually subsided, and at this time—one month after the operation—he enjoys perfect freedom from pain, there being only a loss of sensation and coldness on the inner side of the leg, which will probably remain some time.

